# Identification of BRAF V600E mutation in odontogenic tumors by high-performance MALDI-TOF analysis

**DOI:** 10.1038/s41368-022-00170-8

**Published:** 2022-04-25

**Authors:** Lucrezia Togni, Antonio Zizzi, Roberta Mazzucchelli, Andrea Santarelli, Corrado Rubini, Marco Mascitti

**Affiliations:** 1 Department of Clinical Specialistic and Dental Sciences, Marche Polytechnic University, via Tronto 10, 60126 Ancona, Italy; 2 Department of Biomedical Sciences and Public Health, Marche Polytechnic University, via Tronto 10, Ancona, Italy; 3 Dentistry Clinic, National Institute of Health and Science of Aging, IRCCS INRCA, via Tronto 10, Ancona, Italy

**Keywords:** Diagnostic markers, Mass spectrometry

## Abstract

Odontogenic tumors are rare lesions with unknown etiopathogenesis. Most of them are benign, but local aggressiveness, infiltrative potential, and high recurrence rate characterize some entities. The MAP-kinase pathway activation can represent a primary critical event in odontogenic tumorigenesis. Especially, the BRAF V600E mutation has been involved in 80–90% of ameloblastic lesions, offering a biological rationale for developing new targeted therapies. The study aims to evaluate the BRAF V600E mutation in odontogenic lesions, comparing three different detection methods and focusing on the Sequenom MassARRAY System. 81 surgical samples of odontogenic lesions were subjected to immunohistochemical analysis, Sanger Sequencing, and Matrix-Assisted Laser Desorption/Ionization-Time of Flight mass spectrometry (Sequenom). The BRAF V600E mutation was revealed only in ameloblastoma samples. Moreover, the presence of BRAF V600E was significantly associated with the mandibular site (ρ = 0.627; *P* value <0.001) and the unicystic histotype (ρ = 0.299, *P* value <0.001). However, any significant difference of 10-years disease-free survival time was not revealed. Finally, Sequenom showed to be a 100% sensitive and 98.1% specific, suggesting its high-performance diagnostic accuracy. These results suggest the MAP-kinase pathway could contribute to ameloblastic tumorigenesis. Moreover, they could indicate the anatomical specificity of the driving mutations of mandibular ameloblastomas, providing a biological rational for developing new targeted therapies. Finally, the high diagnostic accuracy of Sequenom was confirmed.

## Introduction

Odontogenic tumors and tumor-like lesions constitute a group of heterogeneous diseases that range from hamartomatous or non-neoplastic tissue proliferation to benign neoplasms to malignant tumors with metastatic potential.^1^ They derive from epithelial, ectomesenchymal and/or mesenchymal elements that still are, or have been, part of the tooth-forming apparatus.^2,3^ They are rare tumors, accounting for less than 1% of all neoplasms. Most of them arise ex novo, although some lesions may originate from pre-existing odontogenic cysts.^2,4^ Most of odontogenic tumors are benign lesion, but local aggressiveness, infiltrative potential, and high recurrence rate characterize some entities. Recurrences can also occur after 15 years, so long-term clinical and radiographic follow-up is required.^5–7^ For several years, studies in the field have focused their interest on the expression of biological behavior markers.^8^ Recently, research showed these lesions can harbor oncogenic alterations already considered as specific tumor drivers in other organs,^9^ boosting the needs for study the molecular mechanisms involved in the development of odontogenic lesions. However, there is no clear overview of these mutations and there are no reliable prognostic markers.^10–12^ Scientific research focused on B-Raf gene mutations, considered the most powerful MAP-kinase (MAPK) pathway activator. It suggests that BRAF V600E mutation represents about 90% of all B-Raf gene mutations, involved in 80–90% of ameloblastic lesions.^10–29^ Therefore, the MAPK activation could contribute to odontogenic tumorigenesis, offering a biological rationale for developing new therapeutic strategies.^9,10^ The role of personalized therapies is still poorly defined, however in vitro and in vivo studies suggest the MAPK pathway as a promising therapeutic target for odontogenic lesions.^16,19,30^ Currently, molecular tests represent the Gold Standard to study the genetic alterations in several types of solid tumors, although highly performing Next Generation Sequencing methods are recently emerged.

The present investigation focuses on the Sequenom MassARRAY System, a highly accurate technology with high specificity and sensitivity in detecting genetic variations in heterogeneous samples.

In addition, its high multiplexing capacity provides to minimize the required sample amount and to maximize the sensitivity.^31–33^ The primary aim of the study is to determine the frequency of BRAF V600E mutation in odontogenic lesions, correlating the mutational status with the clinicopathological and prognostic features. Moreover, the study aims to compare three different BRAF V600E mutation detection methods, focusing on the Sequenom MassARRAY System. The molecular characterization could provide the biological basis for the development of new targeted therapies, improving patients’ treatments.

## Results

### Clinicopathological data of odontogenic lesions

The study included 81 surgical specimens: 48 ameloblastomas, 4 calcifying epithelial odontogenic tumor (CEOT), 19 odontogenic keratocyst (OKC), 5 odontogenic carcinomas, 3 odontogenic clear cell carcinoma (OCCC), and 2 ameloblastic fibrosarcomas. The surgical specimens are related to 46 patients, with a male: female ratio equal to 2.3:1 and mean age at diagnosis of (47.6 ± 20.1) years (range: 8–91 years). The mandible was the most involved site (49 cases), with a mandible:maxilla ratio of 1.5:1. The mean diameter of lesions was (4.5 ± 1.6) cm (range: 0.4–8 cm) and the disease-free survival (DFS) time after the initial surgical treatment was (48.8 ± 36.2) months (range: 4–161 months). Recurrences mainly affected the maxilla (24/35) and the male gender (27 / 35), with a mean age at diagnosis of (50.6 ± 20.2) years. The main clinicopathological features of lesions are shown in Table 1.

**Table 1:** Main clinicopathological features of odontogenic lesions

Clinicopathological data	AMB	UA	AC	A E/P	OKC	CEOT	Malignant OT	Total cases
Number of patients	26	14	10	5	10	3	7	46
Number of lesions	48	24	17	7	19	4	10	81
Mean age	49.2 ± 18.4	40.0 ± 17.4	57.6 ± 13.6	64.0 ± 11.1	38.2 ± 17.9	31.0 ± 20.0	64.5 ± 19.9	47.6 ± 20.1
Gender^a^								
Male	18	10	7	4	8	1	5	32
Female	8	4	3	1	2	2	2	14
Site								
Maxilla	15	7	5	4	9	3	5	49
Mandible	33	17	12	3	10	1	5	32
Size/cm	3.1 ± 1.8	3.6 ± 2.1	3.0 ± 1.6	1.5 ± 0.7	2.4 ± 0.8	3.1 ± 1.9	4.3 ± 1.1	4.5 ± 1,6
Primitive	27	15	9	3	11	2	6	46
Recurrence	21	9	8	4	8	2	4	35
DFS time/months	51.1 ± 41.5	41.4 ± 36.6	41.0 ± 31.4	70.8 ± 9.8	47.0 ± 22.3	23.5 ± 16.3	53.7 ± 37.6	48.8 ± 36.2

*AMB* ameloblastoma, *UA* unicystic ameloblastoma, *AC* conventional ameloblastoma, *A E/P* extraosseous/peripheral ameloblastoma, *OKC* odontogenic keratocyst, *CEOT* calcifying epithelial odontogenic tumor, *OT* odontogenic tumor, *DFS* disease-free survival

^a^The gender distribution is referred to the number of patients

Regarding ameloblastoma, the male was the most affected gender, and the mandible was the most involved site. Half of them were diagnosed as unicystic ameloblastoma (UA) and the mean age at diagnosis was (49.2 ± 18.4) years. The histological pattern was mainly mixed, without differences in its distribution related to the histological variant. Recurrences accounted for 21 cases, with twofold time higher incidence in men and a DFS time of (51.1 ± 41.5) months. The odontogenic keratocysts were related to ten patients with a male:female ratio of 4:1 and a mean age at diagnosis of (38.2 ± 17.9) years. Relapses occurred only in male patients, after (46.3 ± 14.1) months. Malignant odontogenic tumors preferred male patients with a mean age at diagnosis of (64.5 ± 19.9) years. Relapses occurred after (53.7 ± 37.6) months; moreover, the OCCC metastasized after (14.7 ± 14) months.

### Comparative analysis of BRAF V600E mutation detection methods

Immunohistochemical analysis and molecular tests were performed on 81 and 74 surgical samples, respectively. BRAF V600E mutation was reported in 20 cases by immunohistochemistry, in 15 cases using Sanger, and in 19 cases by Sequenom analysis. The BRAF V600E mutation was revealed only in ameloblastoma samples, regardless of the detection method. None of OKC, CEOT, and odontogenic malignant tumors displayed the mutation. Focusing on ameloblastoma, the mutational rate was equal to 42.6% (20/47), 31.9% (15/47), and 40.4% (19/47) using immunohistochemistry, Sanger, and Sequenom, respectively.

**Fig. 1:**
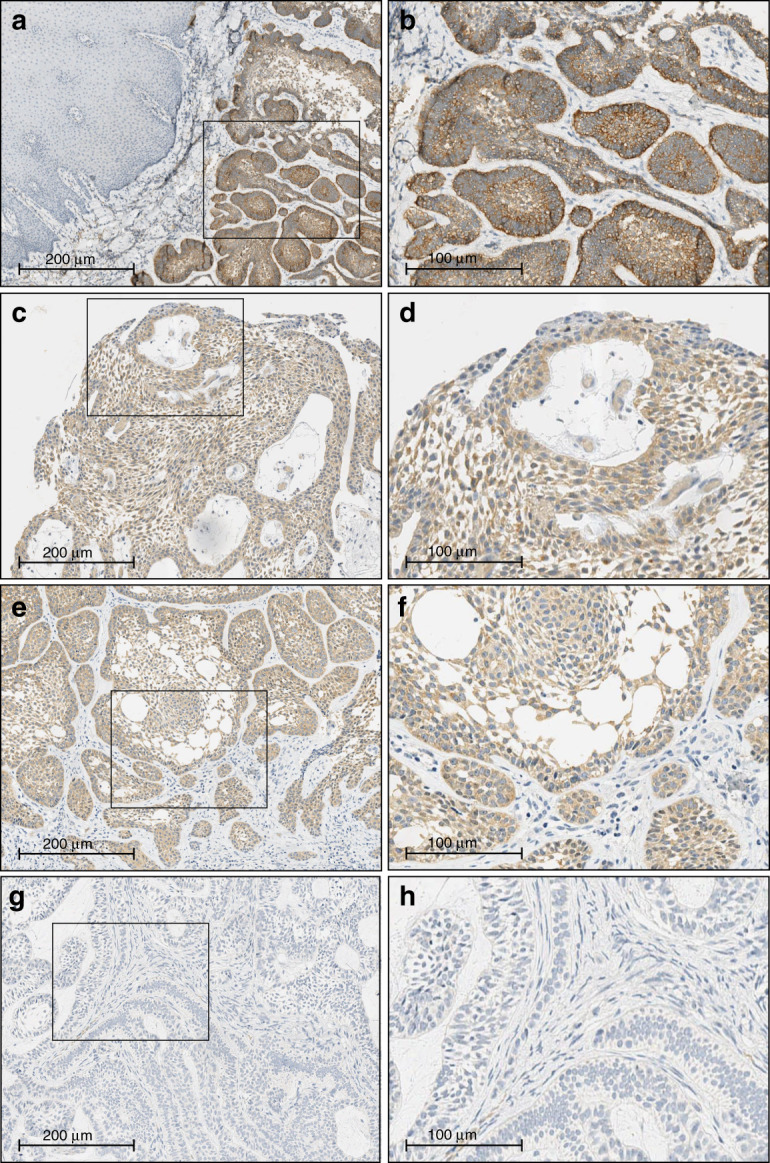
Immunohistochemical expression of BRAFV600E in odontogenic lesions. **a** Peripheral/Extraosseous Ameloblastoma: intense expression of BRAF V600E mutation, both in the follicular and in the plexiform pattern (×2 magnification). The mutation extension involves 30%–40% of the tumoral cells (*n*. 42, Supplementary Table 1). **b** The inset area of greater magnification (×20) shows the cytoplasmic expression of BRAF V600E both in the peripheral layer and in the central cells of neoplastic nests and strands. **c** Unicystic Ameloblastoma: moderate intensity and high extension (90%) of BRAF V600E mutation in plexiform pattern (×10 magnification), (*n*. 7, Supplementary Table 1). **d** The inset area of greater magnification (×20) shows the cytoplasmic expression of BRAF V600E in the ameloblastic epithelium islands. **e** Unicystic Ameloblastoma: moderate intensity and high extension (70%–80%) of BRAF V600E mutation in follicular pattern (×10 magnification), (*n*. 3, Supplementary Table 1). **f** The inset area of greater magnification (×20) shows the cytoplasmic expression of BRAF V600E both in the peripheral layer and in the central neoplastic cells. **g** Conventional Ameloblastoma: absence of BRAF V600E expression (<2%) both in plexiform and in follicular pattern (×10 magnification), (*n*. 25, Supplementary Table 1). **h** The inset area of greater magnification (×20) shows no BRAF V600E expression

On immunostaining, a weak to moderate cytoplasmic reactivity was found both in the peripheral palisading layer and in the central loosely arranged cells of neoplastic nests and strands, whereas no staining was detected in the stromal components and in non-neoplastic tissues (Fig. 1). Regarding histological subtypes, the BRAF V600E mutation mainly involved UA (70.0–78.9%), regardless the detection method. The BRAF protein was uniformly expressed along the full length of the epithelial lining, as well as in the odontogenic epithelium islets scattered throughout the tumor capsule (Fig. 1).

Three and one BRAF wild-type samples (immunohistochemistry and Sanger, respectively) resulted BRAF mutation at Sequenom. Moreover, immunohistochemical analysis revealed the mutation in two molecular wild-type cases. All mutated lesions detecting at Sanger sequencing were confirmed by Sequenom. Furthermore, Sequenom identified other types of mutations involving the MAPK and the phosphatidylinositol-3-kinase (PI3K) pathway. These mutations affected 12.2% of the samples (9/74): five ameloblastomas (10.6%), two odontogenic keratocysts (10.5%), and two malignant odontogenic tumors (20 %). The mutations concerned the RAS (77.8%) and the PIK3CA gene (22.2%). Especially, ameloblastoma expressed the KRAS G12R (n. 11; Supplementary Table 1), NRAS Q61R (n. 38, 41; Supplementary Table 1), and PIK3CA T1025T (n. 28, 29; Supplementary Table 1) mutations. The OKCs hosted the NRAS Q61L mutation (n. 58, 65; Supplementary Table 1), and the odontogenic carcinomas arose the KRAS A146V mutation (n. 73, 74; Supplementary Table 1). Regarding the ameloblastomas, the mutations have been detected only in BRAF wild-type samples (*P* = 0.038). The mutational status is represented in Supplementary Fig. 1.

Regarding the accuracy analysis of ameloblastoma samples a sensitivity of 100% (95% confidence interval [CI]: 97.6%–100%), a specificity of 96.2% (95% CI: 81.1%–99.8%), a Positive Predictive Value (PPV) of 93.8% (95% CI: 71.7%–99.7%), a negative predictive value (NPV) of 100% (95% CI: 86.7%–100%), a positive likelihood ratio (LR) of 26, and a negative LR of 0, were recorded (Supplementary Table 2).

**Fig. 2:**
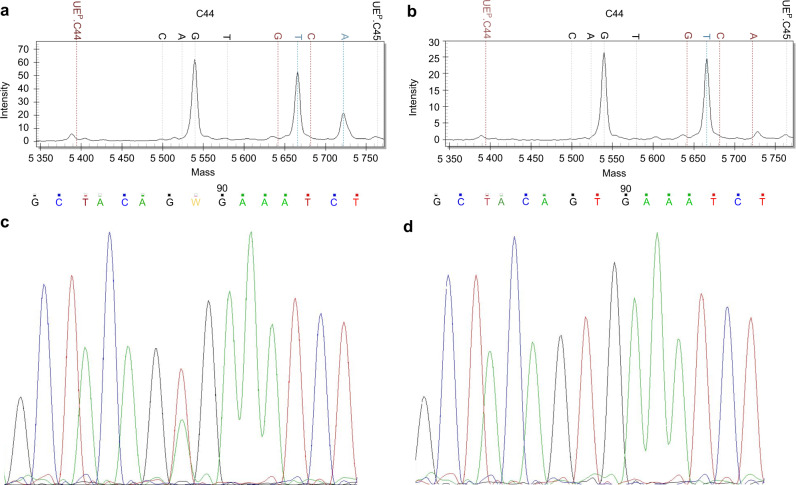
Sanger sequencing and MALDI -TOF analysis of odontogenic lesions. **a** Forward MALDI-TOF Mass Spectrum of a BRAF V600E unicystic ameloblastoma (*n*. 7, Supplementary Table 1). The mutation is characterized by the replacement of thymine with adenine. **b** Forward MALDI-TOF Mass Spectrum of a wild-type unicystic ameloblastoma (*n*. 4, Supplementary Table 1). The adenine and thymine rates are equal to 0 and 23%, respectively. **c** Sanger Sequencing Electropherogram (Codon V600; Exon 15; B-Raf gene) of unicystic ameloblastoma (*n*. 7, Supplementary Table 1). Forward mutation of the BRAF codon encoding p. Val600Glu (V600E), characterized by the substitution of thymine with adenine in position 1799 (c.1799 T > A). The adenine and thymine rates are increased up to 20 and 50%, respectively. **d** Sanger Sequencing Electropherogram (Codon V600; Exon 15; B-Raf gene) of unicystic ameloblastoma. (*n*. 4, Supplementary Table 1). Wild-type allele in forward for BRAF V600E mutation, characterized by the nucleotide sequence encoding for the amino acid Valine, A adenine, C cytosine, G guanine, T thymine

The immunohistochemistry showed to be a 78.6% sensitive and a 94% specific, suggesting that the immunohistochemistry performed on undecalcified tissue sections is a suboptimal surrogate of genetic tests. The mutational status of all samples is summarized on Supplementary Table 1. Examples of ameloblastomas mass spectrums and electropherograms are represented in Fig. 2.

### Correlation between ameloblastoma clinicopathological data and Sequenom mutational status

The 40.4% (19/47) of ameloblastomas harbored the BRAF V600E mutation. Clinicopathological variables were firstly explored by Spearman rank correlation analysis. The presence of BRAF V600E was significantly associated with the mandibular site (ρ = 0.627; *P* value <0.001; Table 2) and the unicystic histotype (ρ = 0.299; *P* value <0.001; Table 2). At Chi-square and Mann–Whitney tests, all the mutated ameloblastomas arose in the mandible and about 80% of cases (15/19) were diagnosed as UA (*P* value <0.0001; Table 3). Contrarily, wild-type ameloblastomas mainly involved the maxillary region (15/25; 60%) and the conventional histological type (14/24; 56%).

**Table 2:** Spearman rank correlation for variables evaluated into the cohort of 44 cases of ameloblastoma

Variable	BRAF V600E	Site	Size	Diagnosis	Recurrence
BRAF V600E	ρ = 1	**0.627**	−0.159	**0.299**	−0.165
	*P* value = 1	**0.000**	0.328	**0.048**	0.283
Site		ρ = 1	−0.237	0.121	−0.279
		*P* value = 1	0.141	0.436	0.066
Size			ρ = 1	−0.030	−0.090
			*P* value = 1	0.852	0.582
Diagnosis				ρ = 1	−0.191
				*P* value = 1	0.214
Recurrence					ρ = 1
					*P* value = 1

Bold values denote statistical significance at the *P* < 0.05

**Table 3:** Differences in clinicopathological data and the presence of BRAF V600E mutation in the cohort of 44 cases of ameloblastoma

Parameter	BRAF V600E	WT	*P* value
Gender			
Male	11	19	>0.05^a^
Female	8	6	
Age	47.1 ± 17.1	50.0 ± 20.5	>0.05^b^
Site			
Maxilla	0	15	<0.000 1^a^
Mandible	19	10	
Histotype			
Conventional	1	14	
Unicystic	15	3	<0.000 1^a^
Peripheral	3	8	
Size (cm)	3.7 ± 2.2	3.2 ± 1.5	>0.05^b^
Clinical form			
Primary	13	13	>0.05^a^
Recurrence	6	12	

*WT* wild type, *cm centimeters*

^a^Chi square test

^b^Mann–Whitney *U* test

Bold values denote statistical significance at the *P* < 0.05

**Fig. 3:**
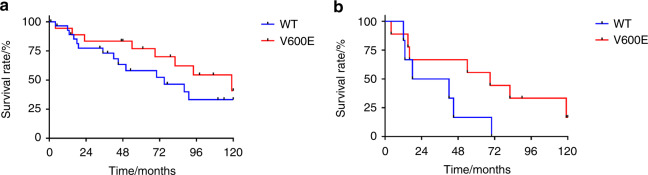
Kaplan–Meier analysis of disease -free survival. **a** Ten-year disease-free survival of patients with or without BRAF V600E mutation (40.8% vs 33.2%). **b** Ten-year disease-free survival of patients affected by unicystic ameloblastoma with or without BRAF V600E mutation (16.7% vs 0%). DFS, disease-free survival; WT, wild type

No significant difference emerged between the presence of BRAF V600E mutation and gender, histological pattern, mean age at diagnosis and mean sizes (*P* value >0.05; Table 3). Both mutated and wild-type samples showed a preference for the male gender, the fifth decade, and the mixed histological pattern. Furthermore, no significant differences in mutational status between the primary and recurrence tumors, were observed. The mutated recurrent tumors arose later than the wild-type relapses, without reaching a significant difference in terms of 10-years DFS (40.8% vs 33.2%: *P* value >0.05) (Fig. 3a).

Regarding UA, Chi-square test showed a statistically significant difference among site of occurrence and the presence of BRAF V600E mutation (*P* value <0.000 1; Table 4). All the mutated UA (15/15) arose in the mandibular region, whereas wild-type UA mainly involved the upper jaw (7/8). Furthermore, there was a different trend between the presence of BRAF V600E mutation and gender (*P* value >0.05). No significant differences between mutational status, histological pattern, mural extension, tumor nature, and lesions size, were observed.

**Table 4:** Differences in clinicopathological data and the presence of BRAF V600E mutation in the cohort of 24 cases of unicystic ameloblastoma

Parameter	BRAF V600E	WT	*P* value
Gender			
Male	9	8	>0.05^a^
Female	6	0	
Age	43.0 ± 16.6	28.0 ± 12.7	>0.05^b^
Site			
Maxilla	0	7	<0.000 1^a^
Mandible	15	1	
Size (cm)	4.0 ± 2.3	3.7 ± 1.5	>0.05^b^
Clinical form			
Primary	11	4	>0.05^a^
Recurrence	4	4	

*WT* wild type, *cm* centimeters

^a^Chi square test

^b^Mann–Whitney *U* test

Bold values denote statistical significance at the *P* < 0.05

The BRAF V600E tumors arose later than the wild-type ones (43.3 ± 16.6 vs 28.0 ± 12.7), without reaching a significant difference. Similarly, the univariate analysis showed a trend between the absence of the BRAF V600E mutation and the onset of relapses.

The mutated recurrences occurred later than wild-type ones (64.3 ± 43.0 vs 28.0 ± 15.5). However, no significant differences for 10-year DFS were found between patients with BRAF V600E mutation and wild-type patients (16.7% vs 0%; *P* value >0.05) (Fig. 3b).

## Discussion

Odontogenic tumors can harbor oncogenic alterations considered specific tumor drivers in other organs. The MAPK pathway mutations, expressed during the dental development, involve 80% of ameloblastic lesions. Especially, the BRAF V600E mutation seems to represent 90% of B-Raf gene mutations, suggesting its role in odontogenic tumorigenesis. However, the rarity of these lesions and the methodological heterogeneity do not ensure sufficient scientific evidence.

We aimed to evaluate the role of BRAF V600E mutation in odontogenic lesions, focusing on the Sequenom MassARRAY System detection method. The first study investigating the presence of the BRAF V600E mutation in ameloblastomas reported a frequency of 62.5%.^34^ Our results displayed an ameloblastoma mutation rate over 40%, in agreement with those reported in literature.^10,11,13–15,17–22,24,26–28,30,35–41^. Our analyses found an association between the presence of the BRAF V600E mutation and the unicystic histotype, in agreement with the literature (72%–100%).^9,17,21,26,36,40,42^ A heterogeneous mutational trend emerged in conventional ameloblastoma, depending on the detection methods used.^9,17,21,24,26,27,30,36–42^ The BRAF V600E mutation was only detected in mandibular ameloblastomas; this association has been demonstrated by others, reporting a high prevalence (82%–100%) of the mandibular localization.^21,24,26,36,40^

Several studies showed a predilection of the lower jaw, without reaching significance.^14,16,19,21–23,42,43^ However, these data should be taken with reserve because some studies did not specify the tumor site.^44^

Sweeney et al. suggested a different etiopathogenesis of ameloblastoma, proposing a molecular-based classification of these lesions, potentially responsive to different targeted therapy: SMO-mutated lesions, typical of early relapsing, maxillary plexiform ameloblastomas, and mandibular BRAF-mutated tumors.^19,24,26^ Our results support this hypothesis which could indicate the anatomic specificity of the driving mutations, suggesting a different developmental signaling pathway.^16,19,24,26,36,40,42,43^

No association emerged between the mutational status and the mean age at diagnosis, although several studies support the early onset of mutated tumors compared to wild-type ones.^14,16,24,26,36,40,43^ Neither association with the tumor pattern was observed, due to the prevalence of mixed histological patterns. However, the literature indicates a prevalence of the plexiform pattern and a uniform histological profile in the wild-type lesions.^10,19,24,40,44^ We reported a lower immunohistochemical BRAF expression in squamous and desmoplastic areas; but in this regard, the literature data are scarce and conflicting.^14,16,40,45^

Mutated recurrences tend to arise later than the wild-type ones.^10,16,19,23,24,26,40,43^ On the contrary, Fregnani et al. demonstrated a significant association between mutational status and aggressive tumor features, including the relapse trend.^14^

The BRAF V600E mutation has also been detected in other ameloblastic lesions. The expression in some ameloblastic carcinomas (25%–100%) could reflect the exclusive positivity in tumors developed from pre-existing ameloblastomas.^13,16,17,41,46^ Therefore, the frequency of BRAF V600E mutation could be negligible in primary ameloblastic carcinoma. Its expression in ameloblastic fibromas (33.3%–100%) could suggest a histological variant of ameloblastoma with similar pathogenesis.^13,16,17,41,46^ There is no evidence of the positivity of other odontogenic lesions^13,15–17,20,25,41,47^ and dental follicles.^48^

Only Cha et al. reported the presence of BRAF V600E in OKC, although immunohistochemistry did not validate their results.^49^ Furthermore, other studies did not attribute any role to the BRAF V600E mutation on the pathogenesis of OKC.^13,16,27,50,51^ Contrary to our results, some Authors have detected the mutation in cases of OCCC and ameloblastic fibrosarcoma.^29,41^

Additional genes of MAPK pathway may be involved in the molecular pathogenesis of odontogenic lesions. In this study, Sequenom detected other mutations belonging to the MAPK and the PI3K pathways. The RAS and the PIK3CA mutations have been identified in 9.5% (7/74) and in 2.7% (2/74) of BRAF wild-type lesions, respectively. In literature, RAS mutations occurred in 20%–53% of BRAF wild-type cases (KRAS: 8%–15%; NRAS: 6%–14%; HRAS: 6%) and some Authors suggest the hypothesis of a mutual exclusivity with BRAF mutation.^10,16,19,20,26,30,52^ Also, PI3K gene mutation has been highly detected in ameloblastomas (66.7%–100%),^53–55^ and its level seems to be correlated to plexiform pattern.^55^

However, other Authors showed additional mutations to B-Raf in ameloblastomas, such as NRAS, HRAS, KRAS, FGFR2, and PIK3CA,^10,23,44^ suggesting they may represent secondary mutations occurring later in the pathogenesis of ameloblastoma.

To “Gultekin et al.,” 21% of ameloblastomas harbored multiple genetic alterations, such as KRAS, PTEN, FGFR2, and PIK3CA, while single NRAS, HRAS, and EGFR mutations occurred only in 5% of BRAF wild-type cases. Plexiform or mixed, and multilocular ameloblastomas seem to be characterized by single NRAS or HRAS mutations, while most follicular ameloblastomas showed multiple gene mutations, suggesting a possible relation with the histological pattern (follicular versus plexiform) and the tumoral behavior (unilocular versus multilocular).^24^

“Gonzalez et al.”, demonstrated multiple mutations only in AC, while AU do not express other mutations besides BRAF V600E, suggesting it could occur in early stages of tumorigenesis and the additional ones could be acquired with tumoral progression. Although multiple mutations are relatively infrequent in ameloblastomas, these would seem to be associated with recurrences. Thus, BRAF V600E ameloblastomas with multiple mutations could acquire several characteristics from the additional mutated genes.^44^ Finally, “Kondo et al.” showed gene and protein expression levels related to KRAS-responsive, EGFR-induced and TGF-B-related genes, tenfold higher in mandibular ameloblastomas, compared to corresponding healthy mucosa samples.^30^

Considering the multiple connections between MAPK and PI3K pathways, and the ERK and MEK overexpression in ameloblastomas, it could be suggested that ERK and MEK activation may be involved in the pathogenesis and growth of ameloblastoma.^44^

The Gold Standard BRAF V600E assessment in metastatic melanomas and papillary thyroid carcinomas is direct DNA sequencing. Immunohistochemistry is a useful test that does not request the DNA extraction and a high tumor cell content, although the epitope antigenicity could be compromised by necrosis and by unsuitable samples preservation. Standard sequencing can analyze samples with >20% mutated tumor cells. Regarding immunohistochemistry, the mutated proteins can be quantitatively lower than the detection limit of the antibody. This issue can be solved detecting the total protein expression, discriminating suitable samples for immunohistochemical analysis.^56^ The disadvantages of each technique make the accuracy and the comparative analyses unreliable. Some authors reported an adequate agreement,^13,16,17,19,22,34,36,38,50^ whereas others showed a high results variability between the detection methods.^15,20,26,29,37,41,49^ Especially, a low sensitivity and a high specificity of immunohistochemistry have been demonstrated.^37,41^ Our results also reported a high specificity and suboptimal sensitivity. The false-negative rate could be attributed to low antibody sensitivity in specific tumors, lack of the BRAF protein despite the presence of the genetic mutation, post-transcriptional modifications or regulatory RNAs affecting the BRAF protein synthesis.^41^ On the contrary, the Sequenom has been proven to be a high-performance accuracy technique. It is more suitable for the detection of single nucleotide and somatic point mutations. Furthermore, it allows to parallel processing of multiple samples in a single multiplexed-PCR reaction, to simultaneously profile hundreds somatic mutations using a single genetic panel, and to detect low-frequency alleles and copy number variations. Finally, it proved to be useful for odontogenic tumors because of it does not require a high DNA amount.

Currently, the surgical treatment represents the elective therapy of odontogenic tumors; however, results from in vitro studies^15,16,19,23,30,34,43,57^ and clinical data^58–63^ suggest the MAPK pathway as a promising therapeutic target of medical treatment. Vemurafenib showed to inhibit the phosphorylation of the BRAF protein on immortalized cell lines of mutated ameloblastoma.^16,19,30^ Furthermore, it drastically reduced the symptoms and the tumor size, ensuring a good tolerance.^61,62^ The intraosseous neoplasm component seems to be less responsive to Dabrafenib than the extraosseous one, due to a primary neoplastic cell resistance.^45^ Therefore, in aggressive lesions, a combined therapy could be recommended.

Three-dimensional organoids of BRAF-mutated ameloblastic epithelial cells showed to self-renewal and selective resist to BRAF inhibitors.^44^ So, the understanding of organoids could represent a pivotal change in the treatment of odontogenic tumors.

In conclusion, Sequenom proved to be a highly accurate method. Given the potential role of targeted therapies in odontogenic tumors, it would be recommendable to integrate the molecular assessment of their mutational status into the routine histopathological diagnostic procedure.

## Materials and methods

The study included 81 surgical samples of primitive and/or recurrent odontogenic lesions, related to 46 patients diagnosed with ameloblastoma, odontogenic keratocyst, calcifying epithelial odontogenic tumor, ameloblastic carcinoma, odontogenic clear cell carcinoma, and ameloblastic fibrosarcoma. The surgical samples were selected from the archive of the Institute of Pathology, Marche Polytechnic University, Ancona, Italy over a period of 25 years (January 1990–December 2015) and matched with their clinicopathological data collected and cataloged from the clinical records. For each patient, the following information were obtained: age, gender, date of birth, date of diagnosis, lesion site, size, histological diagnosis, therapeutic protocol, last follow-up, and recurrence. To confirm the original diagnosis, each sample was histologically re-evaluated and reclassified according to the 4th Edition of the World Health Organization (WHO) Classification of Head and Neck Tumors.^4^ Only patients with minimal clinical-radiographic follow-up of 5 years, complete clinicopathological data, and suitable biological material for an adequate immunohistochemical and molecular evaluation were included.

In all, 4-µm serial sections tissue of representative diagnostic areas, from formalin-fixed, paraffin-embedded (FFPE) blocks, (were carried out from each sample. The selected samples were subjected to immunohistochemical analysis using Ventana anti-BRAF V600E (VE1) mouse monoclonal primary antibody (Roche Diagnostics GmbH, Mannheim, Germany) and to molecular analyses by standard DNA sequencing (Sanger Sequencing; Applied Biosystems, Lincoln Centre Drive, Foster City, CA, USA) and by single nucleotide polymorphisms (SNP) sequencing technique (Sequenom MassARRAY System; Agena Bioscience, Hamburg, Germany; Italian Distributor Diatech Pharmacogenetics, Jesi, Italy).

Informed consent was obtained from all patients. The study was carried out in accordance with the Ethics Codes of the World Medical Association (Declaration of Helsinki) and was approved by the Regional Ethics Committee of Marche (Protocol No. 2020-365).

### Immunohistochemical analysis

Only sections containing sufficient odontogenic epithelium to assess the antibody reactivity were considered for the investigation. Deparaffined and rehydrated sections were pre-treated with the Cell Conditioning 1 (pH 8) for 64 min and a pre-primary antibody peroxidase inhibitor, followed by incubation with undiluted VE1 mouse monoclonal primary antibody (Ventana Medical Systems) for 16 min at 37 °C, using BenchMark ULTRA automated slide stainer. The OptiView DAB IHC Detection Kit (Ventana Medical Systems) was used to detect BRAF protein expression. Tissues were counterstained with Hematoxylin II for 4 min and with Bluing Reagent for 4 min (Ventana Medical Systems). Two lymph node metastases of malignant melanomas, BRAF V600E mutated and wild-type, were used as positive and negative run control slides, respectively. An unambiguous, uniform, cytoplasmic staining, weak to moderate, of viable tumor cells was considered as “positive” staining. A faint diffuse staining, isolated nuclear staining, and a weak staining of single interspersed cells or stromal inflammatory cells were scored as “negative”.^56^

Two expert pathologists (C.R. and R.M.) independently assessed the positivity for BRAF V600E, blinded to the clinicopathological data. Each specimen was analyzed three times, and any disagreement between the two pathologists was settled by consensus.

### BRAF V600E Sanger sequencing

The Sanger sequencing was conducted on the genomic DNA extracted from the area containing the greatest amount of tumor cells, using the QIAamp DSP DNA FFPE Tissue kit (QIAGEN, Chatsworth, USA). A 20% of viable cancer cells were used as the minimum cutoff to avoid false negatives.^52^ The quantification of the extracted genomic DNA was carried out using a Nanodrop 1 000 UV per Vis spectrophotometer (Thermo SCIENTIFIC). The extracted DNA was amplified by Polymerase Chain Reaction (PCR) of exon 15 of the B-Raf gene, in thermal cyclers using the following parameters: forward primer: 5′-TCATAATGCTTGCTCTGATAGGA-3′; reverse primer: 5′‐GGCCAAAAATTTAATCAGTGGA‐3′; Hybridization temperature: 52 °C; Amplified size: 250 bp. The electrophoresis was carried out using 1.8% agarose gel, the TBE (Tris-borate in ETDA) 10× buffer solution and the intercalating Ethidium Bromide. To confirm the amplified size, a mixture of DNA fragments of known length was used. Later, the GelRed dye (Biotium, Hayward, USA) allowed the results visualization by a transilluminator. DNA purification was performed with QIAquick PCR Purification Kit. Then, it was subjected to cycle sequencing, and the sequencing products were detected by the automatic sequencer. After cycle sequencing, the DNA purification was conducted with DyeEx 2.0 Spin Kit (QIAGEN). Sanger sequencing was performed with the ABI PRISM 3130 Genetic Analyzer automatic sequencer and the fluorescence signal processing using Sequencing Analysis 5.3.1 Software (Applied Biosystems). Finally, the raw data were reworked with Sanger Sequence Software 2.0 (Applied Biosystem) and compared in real time with the nucleotide sequence available on the National Centre for Biotechnology Information database by a molecular biologist (A.Z.).

### Sequenom MassARRAY system

The genomic DNA, extracted from the area containing the greater amount of tumor cells, was submitted to a Multiplexed-PCR reaction and to thermal cycling.

The Myriapod^R^ Colon status kit (Diatech Pharmacogenetics, Jesi, Italy) was used. It allows a high-throughput analysis of about 190-point mutations in 4 genes commonly involved in solid tumors, such as colorectal cancer and melanoma. The genes tested for this study were KRAS (codons 12, 13, 59, 61, 117, 146), B-Raf (codons 594, 600, 601), NRAS (codons 12, 13, 18, 59, 61, 117, 146) and PIK3CA (codons 38, 81, 88, 93, 108,118, 345, 420, 539, 542, 545, 546, 549, 1021,1025, 1043, 1047, 1049).

The enzymatic purification was carried out by Shrimp Alkaline Phosphatase. Subsequently, the purified DNA was subjected to the Primer Extension reaction with a specific Iplex Cocktail and thermal cycling. Allele-specific analytes were purified with SpectroCLEAN and transferred to SpectroCHIPS. Their detection was performed with mass spectrometry, MassARRAY Compact MALDI-TOF (Sequenom; Bruker Instruments), using the SpectroREADER mass spectrometer and the MassARRAY TyperAnalyzer 4 flight time analyzer (Agena Bioscience). The SpectroTyper RTTM Software automatically identified the SNP alleles, converted into a nucleotide sequence by a “base calling” process. Finally, the mutational status of each case was validated on OncoFOCUS Panel v3 (Agena Bioscience), by a molecular biologist (A.Z.). The data were automatically saved, in FASTQ format, on the MassARRAY database.

### Statistical analyses

Clinicopathological variables were explored by Spearman rank correlation analysis. Differences among ameloblastoma groups were established by Chi-square and Mann–Whitney tests. Cox’s multivariate analysis was used to evaluate the correlations between mutational status and the clinicopathological data. A *P* value <0.05 was accepted as statistically significant.

Disease-free survival analyses were conducted using the Kaplan–Meier algorithm and the survival curves were compared using the long-rank test. GraphPad Prism version 7.00. was used. The Sequenom accuracy measures were analyzed, and 95% confidence interval were calculated, using Sanger sequencing for reference as the Gold Standard detection method.^56^

The clinical endpoint was the DFS. The follow-up has been calculated from the date of surgical treatment to the disease recurrence, the date of death, or the date of the last visit.

## Supplementary information

text summary of supplemental matherials

Supplemental Figure 1

Supplemental Table 1 v.2

Supplemental Table 2

Supplemental Table 3

## Data Availability

Data are available on request from the authors.

## Code availability

The study was approved by the Regional Ethics Committee of Marche, University-Hospital Company, Ancona General Hospital (Protocol No. 2020-365).
