# Supplementary material for: Identification of BRAF V600E mutation in odontogenic tumors by high-performance MALDI-TOF analysis
Source: Int J Oral Sci. 2022 Apr 25;14:22. doi: 10.1038/s41368-022-00170-8 (PMC9038922; doi:10.1038/s41368-022-00170-8)
Supplement: Supplementary file 1 — text summary of supplemental matherials [file 41368_2022_170_MOESM1_ESM.pdf]

Supplemental Figure 1.

Oncoprint of the mutational status of odontogenic lesions. Distribution of mutated genes regarding to the diagnosis, gender, age, anatomical site, and tumoral status. (AC: conventional ameloblastoma; AU: unicystic ameloblastoma; AEP: extraosseous / peripheral ameloblastoma; CA: ameloblastic carcinoma; OKC: odontogenic keratocyst; CEOT: calcifying epithelial odontogenic tumor; F: female; M: male; MAND: mandible; MAX: maxilla; P: primitive; R: recurrence; WT: wild type).

Supplemental Table 1

Main clinical-pathological data and mutational status of odontogenic lesions, and immunohistochemical expression of BRAF V600E.

Supplemental Table 2

Diagnostic accuracy parameters in ameloblastoma lesions. Comparative analysis between Sequenom MassARRAY System and Sanger sequencing.

Supplemental Table 3

Diagnostic accuracy parameters in odontogenic lesions. Comparative analysis between Sequenom MassARRAY System and Sanger sequencing.
