# Supplementary figures and images for: Identification of BRAF V600E mutation in odontogenic tumors by high-performance MALDI-TOF analysis
Source: Int J Oral Sci. 2022 Apr 25;14:22. doi: 10.1038/s41368-022-00170-8 (PMC9038922; doi:10.1038/s41368-022-00170-8)

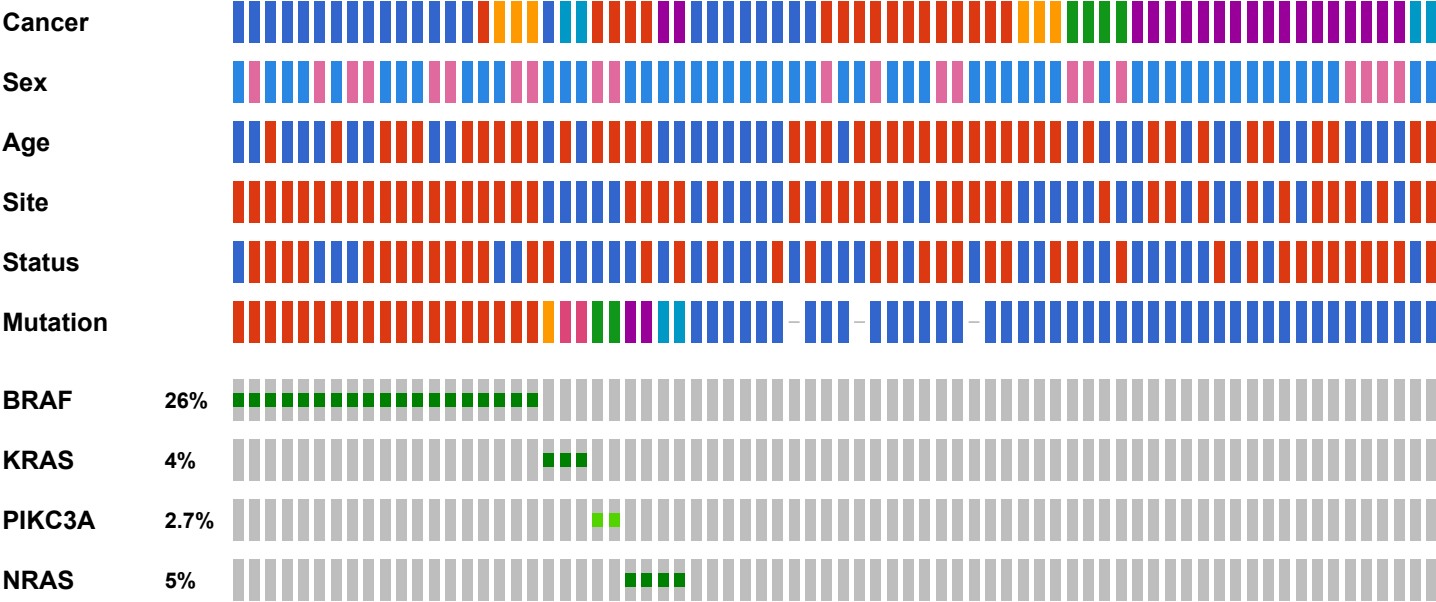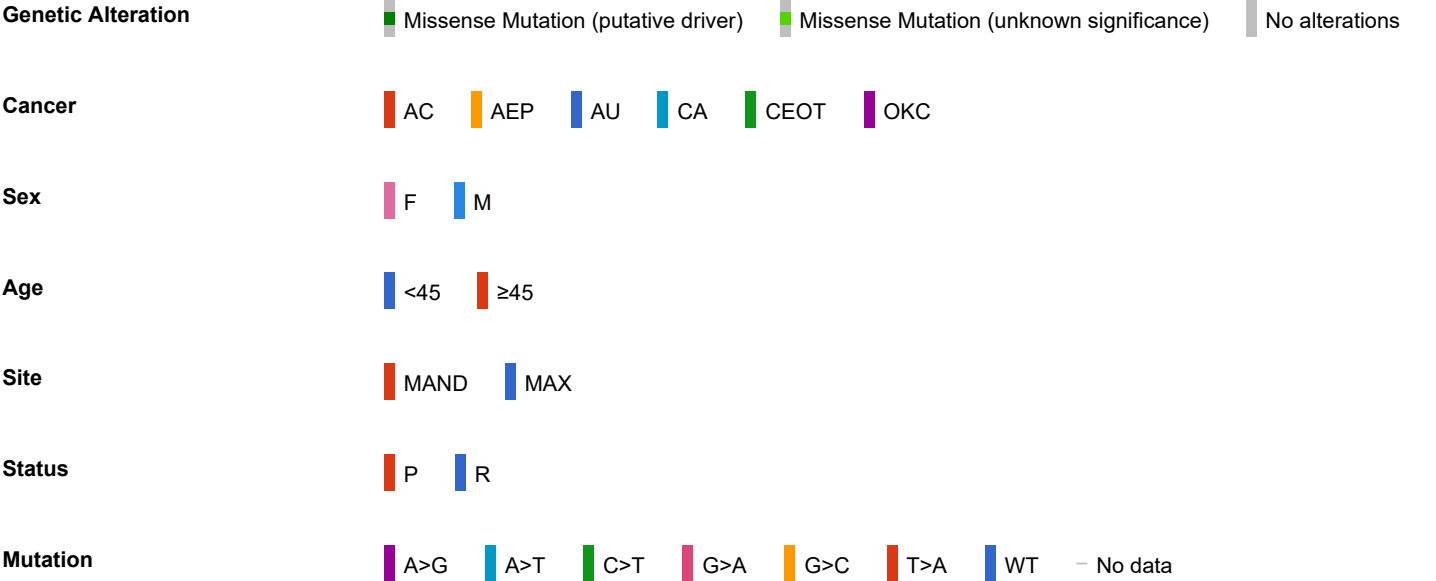

Supplement: Supplementary file 2 — Supplemental Figure 1 [file 41368_2022_170_MOESM2_ESM.pdf]
