# Supplementary material for: Identification of BRAF V600E mutation in odontogenic tumors by high-performance MALDI-TOF analysis
Source: Int J Oral Sci. 2022 Apr 25;14:22. doi: 10.1038/s41368-022-00170-8 (PMC9038922; doi:10.1038/s41368-022-00170-8)
Supplement: Supplementary file 3 — Supplemental Table 1 v.2 [file 41368_2022_170_MOESM3_ESM.pdf]

N*	Diagnosis	Gender	Age	Site	Status	IHC	Sanger	Sequenom
1	AU	M	26	Max	Rec	Neg	WT	WT
2	AU	M	31	Mand	Rec	Pos	BRAF V600E	BRAF V600E
3	AU	F	38	Mand	Prim	Pos	BRAF V600E	BRAF V600E
4	AU	M	32	Mand	Prim	Pos	WT	WT
5	AU	M	62	Mand	Prim	Pos	BRAF V600E	BRAF V600E
6	AU	M	29	Mand	Prim	Pos	BRAF V600E	BRAF V600E
7	AU	M	29	Mand	Prim	Pos	BRAF V600E	BRAF V600E
8	AU	M	22	Max	Rec	Neg	WT	WT
9	AU	M	21	Max	Rec	Neg	WT	WT
10	AU	F	31	Mand	Rec	Pos	BRAF V600E	BRAF V600E
11	AU	M	28	Max	Prim	Neg	WT	KRAS G12R
12	AU	M	22	Max	Rec	Neg	WT	WT
13	AU	M	66	Mand	Rec	Pos	BRAF V600E	BRAF V600E
14	AU	F	44	Mand	Rec	Pos	NA	BRAF V600E
15	AU	F	18	Mand	Prim	Pos	WT	BRAF V600E
16	AU	M	57	Mand	Prim	Pos	BRAF V600E	BRAF V600E
17	AU	M	16	Max	Prim	Neg	WT	WT
18	AU	M	66	Mand	Rec	Neg	NA	NA
19	AU	M	65	Mand	Prim	Pos	NA	BRAF V600E
20	AU	M	60	Mand	Prim	NV	BRAF V600E	BRAF V600E
21	AU	F	38	Mand	Prim	Pos	NA	BRAF V600E
22	AU	F	21	Mand	Prim	Pos	BRAF V600E	BRAF V600E
23	AU	M	56	Mand	Prim	Neg	BRAF V600E	BRAF V600E
24	AU	M	57	Max	Prim	Neg	WT	WT
25	AC	F	56	Mand	Rec	Neg	WT	WT
26	AC	M	33	Mand	Rec	Neg	WT	WT
27	AC	M	49	Mand	Rec	Neg	NA	NA
28	AC	F	68	Max	Rec	Neg	WT	PIKC3A T1025T
29	AC	F	68	Max	Rec	Pos	WT	PIKC3A T1025T
30	AC	F	54	Mand	Prim	Neg	WT	WT
31	AC	M	48	Mand	Prim	Neg	WT	WT
32	AC	M	80	Max	Rec	Neg	WT	WT
33	AC	M	82	Max	Prim	Neg	WT	WT
34	AC	M	64	Mand	Prim	Pos	BRAF V600E	BRAF V600E
35	AC	F	50	Mand	Prim	Neg	WT	WT
36	AC	F	50	Mand	Prim	Neg	WT	WT
37	AC	M	45	Mand	Rec	Pos	NA	NA

38	AC	M	66	Mand	Rec	Neg	WT	NRAS Q61R
39	AC	M	44	Mand	Prim	Pos	WT	WT
40	AC	M	73	Mand	Prim	Neg	WT	WT
41	AC	M	50	Mand	Prim	Neg	WT	NRAS Q61R
42	A E/P	M	59	Mand	Prim	Pos	NV	NV
43	A E/P	M	73	Mand	Rec	Pos	BRAF V600E	BRAF V600E
44	A E/P	M	83	Max	Rec	Neg	WT	WT
45	A E/P	F	60	Mand	Rec	Neg	BRAF V600E	BRAF V600E
46	A E/P	M	67	Max	Rec	Neg	WT	WT
47	A E/P	F	52	Mand	Prim	Neg	BRAF V600E	BRAF V600E
48	A E/P	M	54	Max	Prim	Neg	WT	WT
49	CEOT	F	12	Max	Prim	Neg	WT	WT
50	CEOT	F	52	Max	Rec	Neg	WT	WT
51	CEOT	M	16	Mand	Rec	Neg	WT	WT
52	CEOT	F	44	Max	Prim	Neg	WT	WT
53	OKC	M	40	Max	Rec	Neg	WT	WT
54	OKC	M	56	Mand	Rec	Neg	WT	WT
55	OKC	M	46	Mand	Rec	Neg	WT	WT
56	OKC	M	37	Max	Rec	Neg	WT	WT
57	OKC	M	59	Mand	Rec	Neg	WT	WT
58	OKC	M	24	Mand	Rec	Neg	WT	NRAS Q61L
59	OKC	M	38	Max	Prim	Neg	WT	WT
60	OKC	M	40	Max	Rec	Neg	WT	WT
61	OKC	M	54	Mand	Prim	Neg	WT	WT
62	OKC	M	68	Max	Rec	Neg	WT	WT
63	OKC	M	42	Mand	Prim	Neg	WT	WT
64	OKC	M	33	Max	Prim	Neg	WT	WT
65	OKC	M	19	Mand	Prim	Neg	WT	NRAS Q61L
66	OKC	M	54	Mand	Prim	Neg	WT	WT
67	OKC	M	60	Max	Prim	Neg	WT	WT
68	S-OKC	F	17	Mand	Prim	Neg	WT	WT
69	S-OKC	F	10	Max	Prim	Neg	WT	WT
70	S-OKC	F	20	Mand	Prim	Neg	WT	WT
71	S-OKC	F	8	Max	Prim	Neg	WT	WT
72	Ca. amb	M	59	Max	Prim	Neg	NV	NV
73	Ca. amb	M	45	Max	Rec	Neg	WT	KRAS A146V
74	Ca. amb	M	38	Max	Rec	NV	WT	KRAS A146V
75	Ca. amb	M	91	Mand	Rec	Neg	WT	WT
76	Ca. amb	M	82	Mand	Prim	Neg	WT	WT
77	Ca. CC	F	81	Mand	Rec	Neg	NV	NV

78	Ca. CC	F	80	Mand	Prim	Neg	NV	NV
79	Ca. CC	F	77	Max	Prim	Neg	NV	NV
80	FS amb	M	52	Mand	Prim	Neg	NV	NV
81	FS amb	M	40	Max	Prim	Neg	NV	NV

Supplemental Table 1. Mutational status of odontogenic lesions. AMB: ameloblastoma; AU: unicystic ameloblastoma; AC: conventional ameloblastoma; A E / P: extraosseous / peripheral ameloblastoma; OKC: odontogenic keratocyst; S-OKC: syndromic odontogenic keratocyst; CEOT: calcifying epithelial odontogenic tumor; Ca. amb: ameloblastic carcinoma; FS amb: ameloblastic fibrosarcoma; Ca. CC: clear cells odontogenic carcinoma; IHC: immunohistochemistry; M: male; F: female; Max: maxilla; Mand: mandible; Prim: primitive; Rec: recurrence; Pos: positive; Neg: negative; NA: No available; NV: No evaluated.
