# Supplementary material for: Identification of BRAF V600E mutation in odontogenic tumors by high-performance MALDI-TOF analysis
Source: Int J Oral Sci. 2022 Apr 25;14:22. doi: 10.1038/s41368-022-00170-8 (PMC9038922; doi:10.1038/s41368-022-00170-8)
Supplement: Supplementary file 4 — Supplemental Table 2 [file 41368_2022_170_MOESM4_ESM.docx]

| Sequenom | Sanger | | |
| --- | --- | --- | --- |
|  | BRAF V600E | BRAF WT | Total |
| BRAF V600E | 15 | 1 | 16 |
| BRAF WT | 0 | 25 | 25 |
| Total | 15 | 26 | 41 |

Supplemental Table 3. Diagnostic accuracy parameters in ameloblastoma lesions. Comparative analysis between Sequenom MassARRAY System and Sanger sequencing. Sensitivity 100% (95% CI: 97.6-100%), Specificity 96.2% (95% CI: 81.1-99.8%), Positive Predictive Value 93.8% (95% CI: 71.7-99.7%), Negative Predictive Value 100% (95% CI: 86.7-100%), Positive Likelihood ratio 26, Negative Likelihood ratio 0.

WT, wild type.
