# Supplementary material for: Identification of BRAF V600E mutation in odontogenic tumors by high-performance MALDI-TOF analysis
Source: Int J Oral Sci. 2022 Apr 25;14:22. doi: 10.1038/s41368-022-00170-8 (PMC9038922; doi:10.1038/s41368-022-00170-8)
Supplement: Supplementary file 5 — Supplemental Table 3 [file 41368_2022_170_MOESM5_ESM.docx]

| Sequenom | Sanger | | |
| --- | --- | --- | --- |
|  | BRAF V600E | BRAF WT | Total |
| BRAF V600E | 15 | 1 | 16 |
| BRAF WT | 0 | 52 | 52 |
| Total | 15 | 53 | 68 |

Supplemental Table 2. Diagnostic accuracy parameters in odontogenic lesions. Comparative analysis between Sequenom MassARRAY System and Sanger sequencing. Sensitivity 100% (95% CI: 79.6-100%), Specificity 98.1% (95% IC: 90-99.9%), Positive Predictive Value 93.8% (95% CI: 71.7-99.7%), Negative Predictive Value 100% (95% CI: 93.1-100%), Positive Likelihood ratio 53, Negative Likelihood ratio 0.

WT, wild type.
